# Immunomagnetic isolation of circulating melanoma cells and detection of PD-L1 status

**DOI:** 10.1371/journal.pone.0211866

**Published:** 2019-02-08

**Authors:** Joseph W. Po, Yafeng Ma, Bavanthi Balakrishna, Daniel Brungs, Farhad Azimi, Paul de Souza, Therese M. Becker

**Affiliations:** 1 Centre for Circulating Tumor Cell Diagnostics & Research at the Ingham Institute for Applied Medical Research, Liverpool NSW, Australia; 2 Western Sydney University, School of Medicine, NSW, Australia; 3 University of New South Wales, South Western Sydney Medical School, Liverpool NSW, Australia; 4 Liverpool Hospital, Liverpool NSW, Australia; 5 Illawarra Cancer Centre, Wollongong Hospital, Wollongong, Australia; University of Queensland Diamantina Institute, AUSTRALIA

## Abstract

Personalised medicine targeted to specific biomarkers such as BRAF and c-Kit has radically improved the success of melanoma therapy. More recently, further advances have been made using therapies targeting the immune response. In particular, therapies targeting the PD-1/PD-L1 or CTLA-4 axes alone or in combination have shown more sustained responses in 30–60% of patients. However, these therapies are associated with considerable toxicities and useful biomarkers to predict responders and non-responders are slow to emerge. Here we developed a reliable melanoma circulating tumor cell (CTC) detection method with PD-L1 evaluation on CTCs. A set of melanoma cell surface markers was tested as candidates for targeted melanoma CTC isolation and a melanoma specific immunostaining-based CTC identification protocol combined with PD-L1 detection was established. In vitro testing of the effect of exposure to blood cells on melanoma cell PD-L1 expression was undertaken. Immunomagnetic targeting isolated melanoma CTCs in up to 87.5% of stage IV melanoma patient blood samples and 3 8.6% of these had some PD-L1 expressing CTCs. Our in vitro data demonstrate PD-L1 induction on melanoma cells in the blood.This study established a robust, reliable method to isolate melanoma CTCs and detect expression of PD-L1 on these cells.

## Introduction

Improved technology for the capture of circulating tumor cells (CTCs) is increasing the utility of CTCs to predict prognosis and patient survival. CTCs are a non-invasive biosource for molecular biomarker detection that can inform precision therapy and together with analysis of circulating tumor nucleic acids (ctRNA and ctDNA) are emerging with high potential for widespread clinical utility (reviewed by [1–3]).

One challenge for biomarker testing from common tissue biopsies is tumor heterogeneity. It is now widely accepted that a single tissue biopsy is poorly representative for a patient’s cancer. This is particular relevant in advanced malignancies, where biopsies of the primary tumor provide limited information at a time of therapy resistance and tumor progression [4]. CTCs have been shown to accurately reflect tumor heterogeneity [5, 6]. Since blood draws can be performed repeatedly during disease progression, they are well suited to identifying emerging resistance mechanisms and monitor treatment response. Blood biopsies offer the opportunity to analyse both ctDNA and CTCs for biomarkers. ctDNA analysis is more sensitive for mutation analysis and easier to perform; CTC analysis provides characterisation of cellular heterogeneity and cell specific expression of RNA or proteins [5, 7–10].

In keeping with this paradigm, CTC isolation should be efficient and include heterogenous populations of cancer cells. Currently most carcinoma CTCs are isolated using capture and identification methods targeted to the epithelial cells. However, these CTC detection strategies cannot be utilized for certain malignancies including melanoma [11–14].

A challenge in melanoma is marked heterogeneity in gene expression leading to altered expression of proteins targetable for CTC isolation or identification. Thus, targeting multiple cell surface proteins for isolation and identification may be better suited for optimal melanoma CTC detection [15, 16].

Systemic treatment of melanoma, has recently undergone revolutionary changes with the discovery of predictive tumor biomarkers, such as BRAF, which predict the efficacy of targeted therapy with small molecule inhibitors such as vemurafinib, or dabrafenib. Remarkable responses are restricted to tumors with the relevant mutations and limited, with resistance inevitably developing with only 6–7 month progression free survival [17, 18]. More recently, immune checkpoint inhibition (ICI) using antibodies directed at either the programmed cell death protein 1 (PD-1), its ligand (PD-L1) or CTLA-4, alone or in combination, has dramatically improved the outcome of metastatic melanoma. Approximately 30–60% of patients respond to drugs like nivolumab alone or in combination with ipilimumab [19, 20]. Combination immunotherapy enhances response rates but results in greater systemic toxicity. In the Checkmate 067 trial combining nivolumab with ipilimumab resulted in 59% grade 3–4 toxicity compared with 21% nivolumab and 28% with ipilimumab alone [19]. Hence, it is highly important to develop mechanisms to identify likely responders to these efficacious but toxic therapies. While expression of PD-L1 in the tumor tissue is currently employed as biomarker for predicting patient response to PD-1 inhibition, it remains controversial and is not part of routine testing in melanoma as significant proportions of patients with PD-L1 negative melanomas have shown treatment response [21–23]. In addition, testing for PD-L1 requires tumor samples, which should ideally be taken shortly before therapy commencement and be longitudinally available to monitor changes and response. While this is challenging for tumor tissue biopsies it is realistic for CTCs.

The aim of the current study is to demonstrate that screening PD-L1 from liquid biopsies (CTCs) is feasible with the use of an efficient protocol to isolate melanoma CTCs. We also present *in vitro* data suggesting that melanoma cell PD-L1 levels are increased when these cells are in blood.

## Materials and methods

### Patients

Fourteen patients with stage IV metastatic melanoma were recruited from Liverpool and Wollongong Hospitals, Australia (Table 1). The study was undertaken with written patient consent and approval of the South Western Sydney Biosafety Committee (HREC/13/LPOOL/158). Per blood draw, 3x 9ml EDTA vacutube (Greiner Bio-One, Frickenhausen, Germany) blood samples were taken.

**Table 1:** Patient cohort.

Clinical Characteristic	n	%
Patient Median Age* 63 (47–88)	**14**	**100**
**Gender**		
Male	9	64%
Female	5	36%
Stage IV subclass*		
M1a/M1b	6	43%
M1c	7	50%
Unknown	1	7%
**Prior Therapies**		
0–1	13	93%
≥2	1	7%
**Treatment** #		
ICI	10	71%
Targeted therapy (BRAF/MEK)	4	29%
Other/Unknown	1	7%

ICI = immune checkpoint inhibitors,

* at diagnosis,

# at time of CTC sample

### Cell lines

SkMel28, A375 (ATCC, in Vitro Technologies, Lane Cove West, Australia), 501mel (kindly provided by Colin Goding), WMM1175 (kindly provided by Graham Mann), MelRM, NM176, MelMS (kindly provided by Peter Hersey) and M230 (kindly provided by Antony Ribas) melanoma cells were maintained in DMEM / 10% FCS, WME-099 lymphocytes were maintained in RPMI / 10% FCS at 37C with 5% CO_2_ enriched atmosphere (S1 Table). All cell lines were small tandem repeat (STR) authenticated (AGRF, Melbourne, Australia).

### Cell surface marker immunocytostaining on cultured cells

Melanoma cells were seeded at 3x10^4^ cells per well in 12-well plates on sterile cover slips. 24h post seeding attached cells were fixed and probed with primary antibody (S2 Table) and AlexaFluor488 conjugated goat-anti-mouse secondary antibody (Life Technologies, Mulgrave, Australia, 1:3000) and mounted with Prolong Antifade containing DAPI (Invitrogen, Carlsbad, USA).

### Flow cytometry

Approximately 5x10^4^ cells were pelleted after detachment from tissue culture flasks using 0.2mM EDTA in PBS to preserve cell surface proteins. Cells were washed with PBS, blocked with 10% FCS/PBS and incubated with the relevant primary antibodies or matched IgG controls (S2 Table) in 10% FCS/PBS. After PBS washes the cells were probed with AlexaFluor488 goat-anti-mouse secondary antibody (1:3000, Life Technologies), washed once before suspension in 300μl PBS. Cells were detected using a FACS-Canto II (Becton Dickinson, North Ryde, Australia).

### Melanoma cell identification antibodies

Initial testing was performed by immunocytostaining as outlined above using the rabbit derived antibodies against human Melan A (clone EP1422Y, LSBio, Seattle, USA, 1:300), S100-β (clone EP1576Y, Abcam, Melbourne, Australia, 1:300) and Gp100 (clone Ep4863(2), Abcam, 1:600) with secondary FITC anti rabbit antibody. Inclusion of healthy donor peripheral blood mononuclear cells (PBMCs) discounted antibody interactions with blood cells. Fluorescently conjugated anti-human GP100 [AlexaFluor488] (clone DT101/BC199/HMB4, Novus Biologicals, Littleton, USA; 1:100), anti-human Melan-A [AlexaFluor488] (clone SPM555, Novus Biologicals, 1:100), anti-human S100-β [FITC] (clone 4C4.9+S100B/1012, Novus Biologicals, 1:500) were obtained for ease of CTC identification staining, throughout the manuscript referred to as Mel-ID staining.

### Spiking of cultured melanoma cells

Cultured cells were detached using 0.2mM EDTA in PBS, counted using a hemocytometer and a suspension of 5x10^3^ cells per ml was prepared. Cell concentration was confirmed by counting cells in 4 independent 5 μl aliquots placed on a slide. 20 μl cell suspension was spotted on an input control slide as well as independently spiked into 3 parallel 9 ml blood (or pre-enriched PBMC) samples per healthy donor and cell line, followed by another input control slide. Input control slides were air-dried and cells counted after Hoechst staining. Only experiments with less than 5% deviation between first and second input control cell numbers were included in the analysis with the input number defined as the average of both input control counts.

### Melanoma cell isolation

Selected antibodies were conjugated to immunomagnetic beads (IsoFlux rare cell enrichment kit, Fluxion, San Francisco, USA) according to the supplier’s instruction (S2 Table). Magnetic beads conjugated to individual (30μl per sample) or a combination of antibodies (30μl each per sample) were used to capture cultured melanoma cells spiked in defined numbers into 10^6^ healthy donor PBMCs. Cells were recovered using the IsoFlux CTC isolation platform (Fluxion) with the standard isolation protocol. After immunostaining for CTC (Mel-ID), CD45 (Alexa Fluor 647 conjugated anti-CD45 clone HI30, Novus Biologicals; 1:200) and Hoechst (Fluxion), imaging was performed using the IX71 fluorescent microscope (Olympus, Tokyo, Japan).

### Immunodetection of melanoma CTCs

Melanoma CTCs from 3x9ml metastatic melanoma patient blood samples were captured in the same way and first probed for CD45 and with the rabbit anti-human-PD-L1 PD-L1 antibody (clone: E1L3N, Cell Signalling Technology, Massachusetts, USA; 1:50), then with Alexa Flour 555 conjugated goat-anti-rabbit secondary antibody (Life Technologies; 1:2000), followed by permeablisation with 0.2% Triton X-100 and Mel-ID probing. Hoechst dye was included in the mounting media.

### PD-L1 modulation on melanoma cells in blood

MelRM, NM176 and SkMel28 cells were spiked into 3x 9ml blood samples from the same blood draw of 3 (for MelRM) or 6 (for NM176 and SkMel28) healthy donors and on input control slides as described above. Cells from blood samples were recovered by using combined targeting of MCAM and MCSP for immunomagnetic “CTC” isolation after 0, 24 and 48 hour storage at room temperature. Samples were stained as per immunodetection of melanoma CTCs including PD-L1 detection. Cells were enumerated by fluorescent microscopy.

## Results

### Cell surface proteins for melanoma CTC isolation

We tested expression of a range of cell surface antigens on a cohort of eight heterogeneous melanoma cell lines to identify possible targets for immunomagnetic cell isolation of melanoma CTCs (S1 Table).

**Fig 1:**
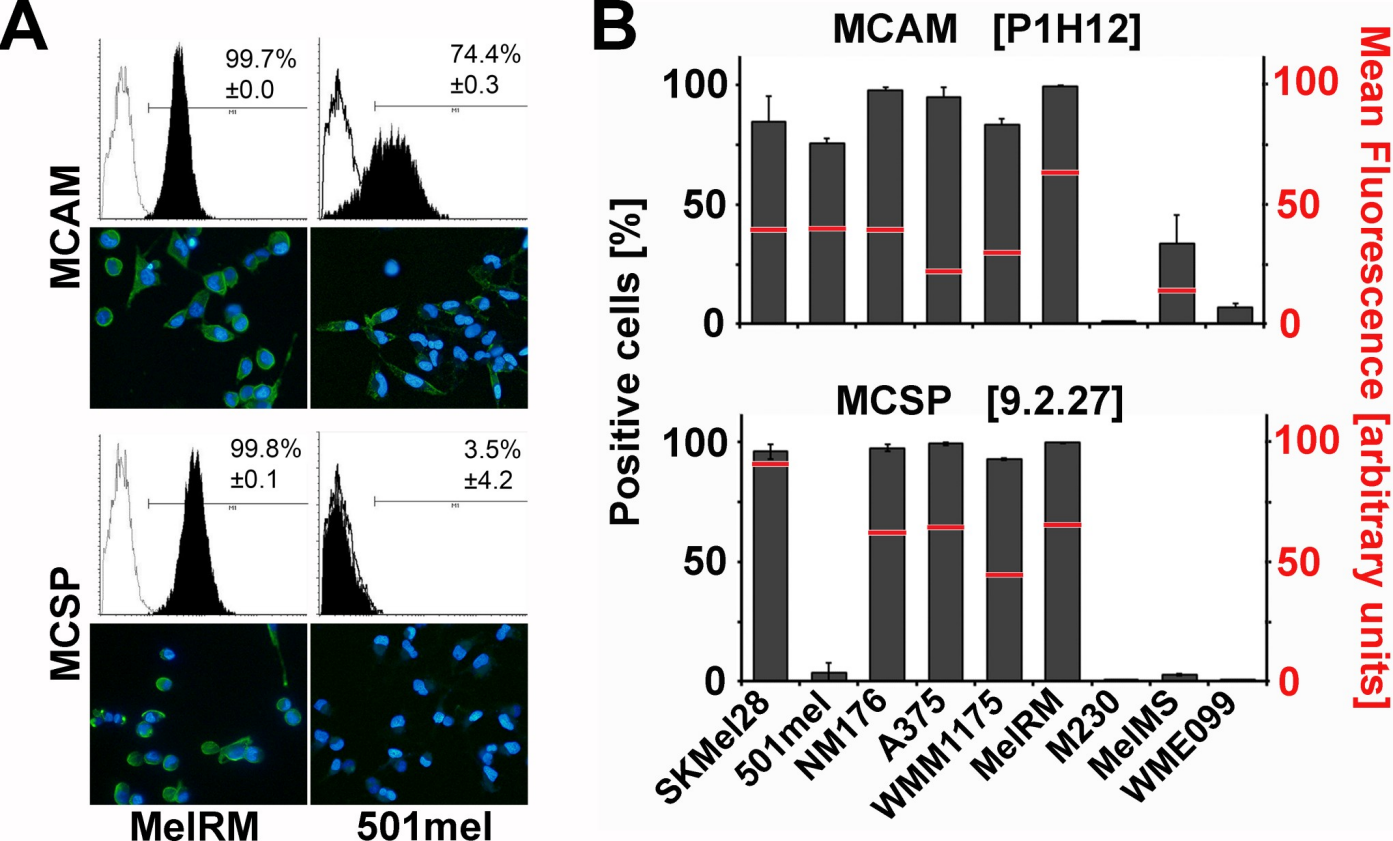
MCAM and MCSP are the dominant melanoma cell surface markers. **(A)** Representative FACS histograms and immunocytostaining for MCAM and MCSP presented for MelRM (left panel) and 501mel (right panel) cells. **(B)** Compiled data for all indicated cell lines showing proportion of cells expressing MCAM and MCSP as detected with the indicated antibodies, data and error bars are derived from three independent experiments. Mean fluorescence (by FACS analysis) is included as a red bar and indicates the antigen expression level on positive cells.

All but two c-Kit mutant cell lines expressed MCAM strongly on 70–100% of cells, irrespective of the anti-MCAM antibody used. MCSP expression was even more pronounced on the melanoma cell lines. Five of eight cell lines expressed MCSP strongly in nearly 100% of the cell populations. However, the BRAF^V600E^ mutant 501mel cells, and both c-Kit mutant lines, were MCSP negative (Fig 1). None of the other cell surface antigens remained convincing candidates for cell isolation due to inadequate expression on the majority of cells. (S1 Fig).

### Melanoma markers for CTC identification

The combination of three antibodies, anti-Melan A, -S100β and -Gp100 detected all melanoma cell lines well (S3 Table) and showed negligible interaction with PBMCs. Directly fluorescently conjugated antibodies against these markers confirmed adequate detection in representative cell lines (Table 2).

**Table 2:** Melanoma CTC identification marker detection in cell lines.

Cell Line	Melan-A *	S100β #	GP100 *	All
SkMel28	**+**	**+++**	**++**	**+++**
501Mel	**++**	**+/-**	**+++**	**+++**
WMM1175	**+**	**+/-**	**++**	**++**
MelRM	**+/-**	**++**	**++**	**++**
PBMCs	**-**	**-**	**-**	**-**

+++ very strong in all cells, ++ strong in ≥85% of cells, + clearly detectable in most cells, +/- clearly detectable in ≥40% of cells, - undetectable

* AF488,

# FITC conjugated antibodies.

### Isolation of melanoma cells

**Fig 2:**
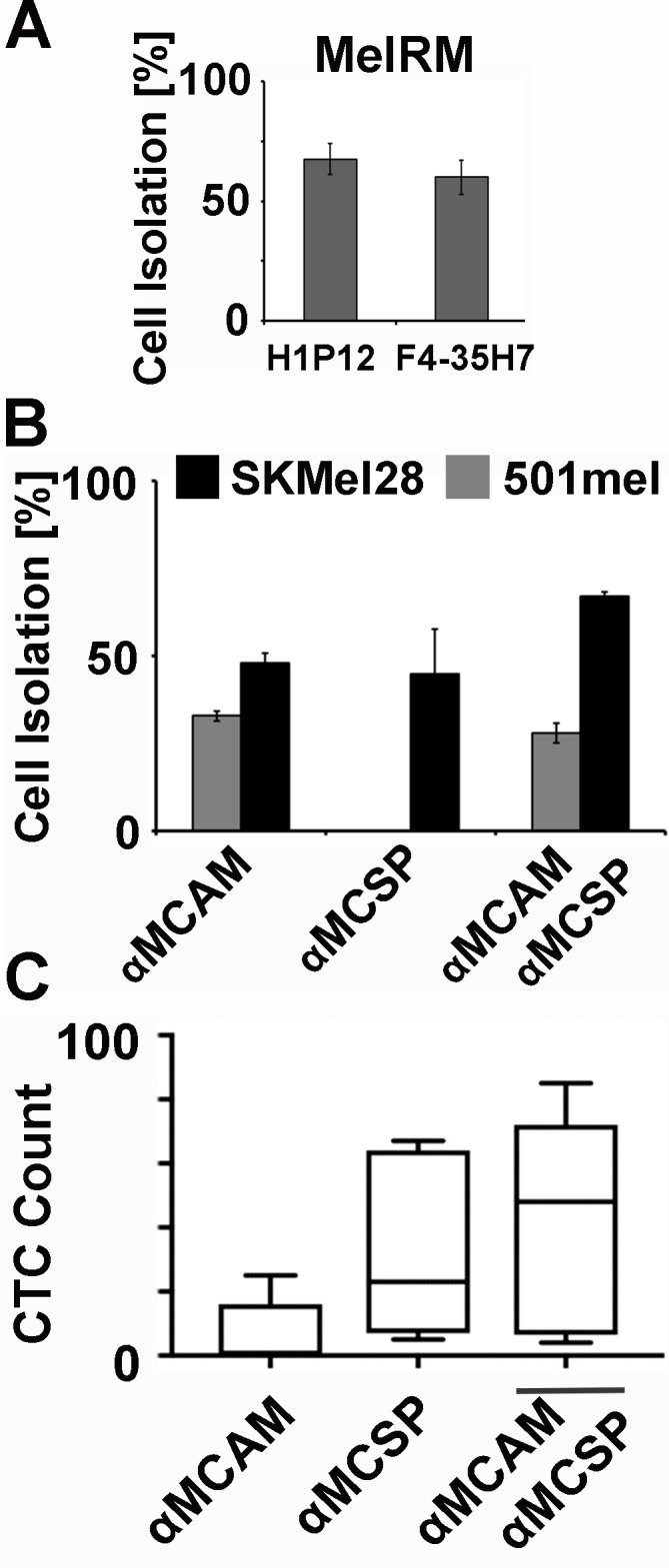
Immunomagnetic melanoma cell isolation. The indicated melanoma cells (n = 100) were spiked into pre-enriched PBMCs in triplicate experiments and then recovered using immunomagnetic beads (Rare Cell Kit, Fluxion) coupled to either **(A)** anti human MCAM antibody clone H1P12 or F4-35H7 or **(B)** to anti human MCAM antibody clone H1P12 (αMCAM) or anti human MCSP antibody clone 9.2.27 (αMCSP) or with the combination of both. The proportion of isolated melanoma cells is presented. **(C)** 14 advanced melanoma patients (16 blood draws, 3x9ml each) were compared for CTC counts after αMCAM (H1P12) based, αMCSP (9.2.27) based or combination based CTC isolation. CTC counts are graphed as box blot.

Initially it was confirmed that both MCAM antibody clones (P1H12 and F4-35H7) behaved similar in isolating MelRM from healthy donor PBMCs (Fig 2A). Comparison of melanoma cell capture using anti-MCAM (clone P1H12), anti-MCSP (clone 9.2.27) revealed cells expressing both proteins (SKMel28) were isolated with either antibody. Combining the two antibodies for isolation improved cell recovery. Expectedly, 501mel cells, which do not express MCSP, were only isolated by MCAM targeting (Fig 2B).

### Melanoma CTC detection from advanced melanoma patients

When comparing CTC isolation from three parallel 9ml blood samples per patient, immunomagnetic targeting of MCAM isolated melanoma CTCs from 62.5% (10/16), MCSP from 81.3% (13/16) and combined MCAM and MCSP targeting from 87.5% (14/16) of samples with median CTC counts of 2.5, 9 and 16 respectively (Table 3 and Fig 2C).

**Table 3:** Melanoma CTC counts and PD-L1 positive CTCs.

						Isolation antibodies			
Patient	Gender	Age#	Stage#	Therapy	Status	αMCAM (PD-L1+)	αMCSP (PD-L1+)	αMCAM / αMCSP (PD-L1+)	combined (27ml blood draw)
1	F	53	M1a	Targeted	N/A	13 (*)	0 (*)	17 (*)	30 (*)
2	M	47	M1c	Immuno	N/A	0 (*)	0 (*)	0 (*)	0 (*)
3	M	56	M1a	Immuno	PR	40 (0)	43 (2)	146 (0)	229 (2)
3^2^	M	56	M1a	Immuno	PR	0 (0)	23 (0)	9 (0)	32 (0)
4	M	60	M1a	Targeted	CR	10 (2)	18 (1)	25 (10)	53 (13)
4^2^	M	60	M1a	ImmunoC	CR	0 (0)	61 (0)	85 (0)	146 (0)
5	M	55	M1c	Targeted	N/A	2 (0)	8 (0)	15 (0)	25 (0)
6	F	62	M1c	Immuno	PR	0 (0)	5 (0)	4 (0)	9 (0)
7	F	70	M1c	Immuno	Prog	7 (0)	9 (0)	48 (0)	64 (0)
8	M	61	M1b	Immuno	PR	25 (0)	67 (1)	59 (0)	151 (1)
9	M	88	M1c	Immuno	S	26 (0)	8 (0)	49 (0)	83 (0)
10	F	64	M1c	Immuno	CR	10 (0)	18 (0)	9 (2)	37 (2)
11	M	78	M1a	Targeted	Prog	2 (0)	9 (0)	43 (0)	54 (0)
12	F	75	M1c	Immuno	Prog	0 (0)	0 (0)	0 (0)	0 (0)
13	M	85	M1a	Immuno	Prog	3 (0)	17 (0)	8 (0)	28 (0)
14	M	64	N/A	N/A	N/A	0 (0)	2 (1)	5 (3)	7 (4)

# At diagnosis, PR: partial response, CR complete response, Prog: progression, S: stable, N/A: data not available,

* no PD-L1 staining done,

Immuno: PD-1/PD-L1 inhibitor, ImmunoC: PD-1 combined with CTLA-4 inhibitor, superscript^2^: repeat sample at later treatment timepoint, αMCAM: anti MCAM antibody [P1H12]; αMCSP: anti-MCSP antibody [9.2.27]

### Detection of PD-L1 on melanoma CTCs

**Fig 3:**
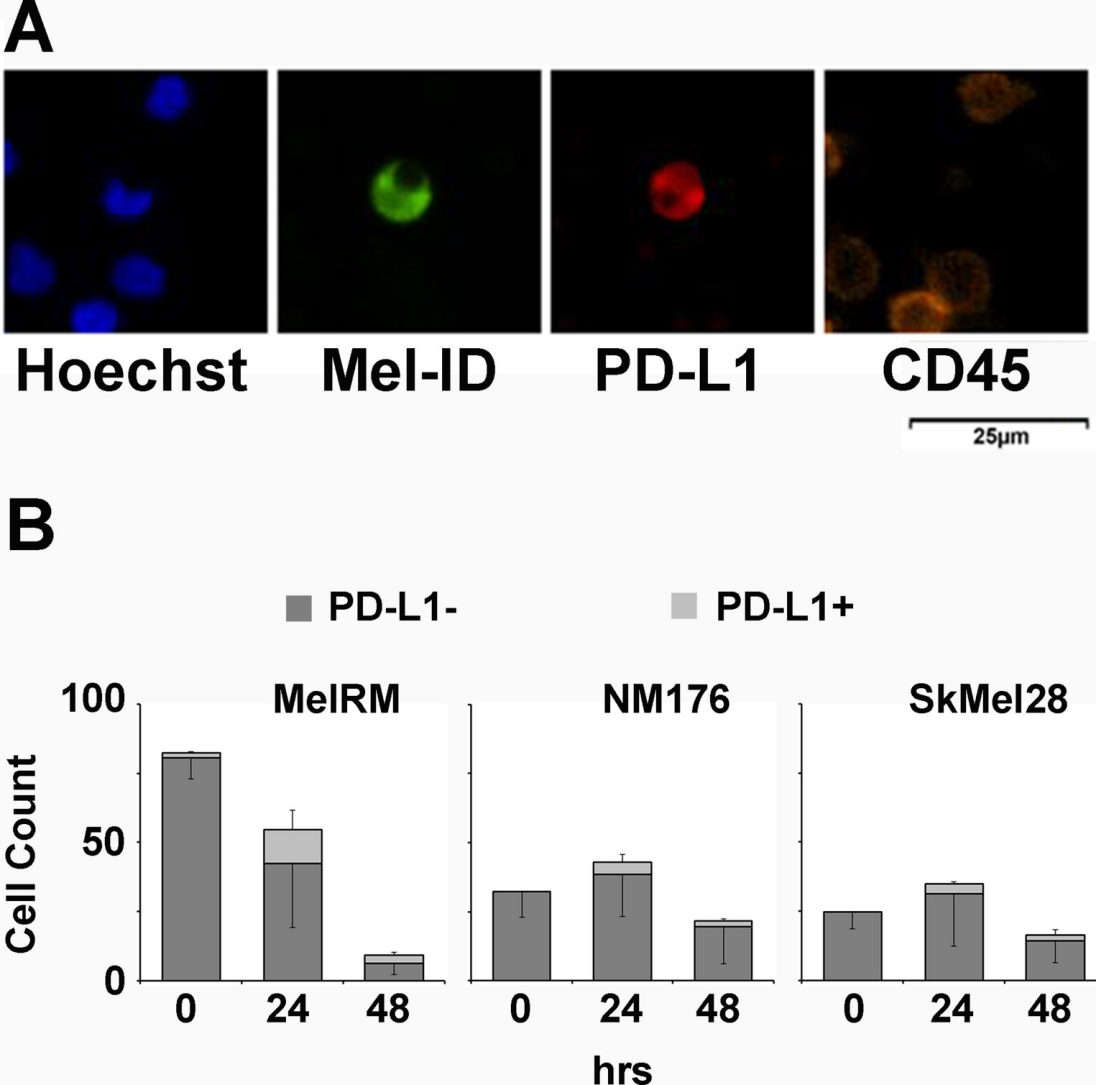
PD-L1 on melanoma CTCs. **(A)** Representative melanoma CTC identification staining with PD-L1 detection of a patient derived CTC surrounded by lymphocytes. **(B)** MelRM, NM176 or SKMel28 cells were spiked into blood samples of healthy donors and isolated immediately or after room temperature blood storage for the indicated time before recovery using our melanoma CTC isolation and immunostaining protocol including PD-L1 probing. Data from three to six experiments (different healthy donors) were analysed per cell line. Mel-ID: probed with cocktail of three fluorescently conjugated melanoma identification antibodies.

As shown in Fig 3A, PD-L1 immunodetection is compatible with melanoma CTC identification probing. Since tissue based cancer cell PD-L1 expression tends to be associated with tumor infiltrating lymphocytes (TILs) [21, 24], we postulated that cancer cell exposure to lymphocytes in the blood, as is the case for CTCs, may trigger adaptive PD-L1 expression in melanoma CTCs.

MelRM and SKMel28 cells, known to be IFN-γ inducible for PD-L1 expression [25] as well as NM176 melanoma cells without previously reported data regarding PD-L1 inducibility, were spiked into healthy donor blood and recovered using the melanoma CTC isolation protocol after room temperature storage for 0, 24 or 48 hours. While for MelRM longer delay until CTC isolation, as expected, reduced melanoma cell recovery, a higher proportion of melanoma cells had detectable PD-L1 after storage. After 24 hours, average cell recovery had dropped by about 50%, but the total number of PD-L1 positive cells had increased and made up about a third of recovered MelRM cells. Although this increased to approximately 50% by 48 hours, overall MelRM recovery was unacceptably low by then. For SKMel28 and NM176 cells no PD-L1 was expressed at baseline, but was detected on >10% of cells after 24 or 48 hours in blood. Interestingly for both these cell lines total cell recovery seemed to slightly, but not significantly increase post 24, while only dropping after 48 hours (Fig 3B).

Finally, we probed for PD-L1 on melanoma CTCs isolated from melanoma patients. For this measure, CTCs, isolated using the various antibodies from the three parallel bloods, were considered as one 27ml blood draw. PD-L1 positive CTCs were detected in 38.6% (5/13) of CTC positive blood draws. In total 1.5–60% of detected CTCs were PD-L1 positive (range of 0–13) (Table 3).

Most of the patients analysed in this study were already on ICI treatment for various cycles and the small, heterogeneous patient cohort, although sufficient for CTC isolation method validation, showed no association of CTC or PD-L1+ CTC counts with clinical parameters, including response, stable disease or disease progression (Table 3).

## Discussion

### Melanoma CTC isolation

The most comprehensive previous investigation of melanoma antigens suitable for immunomagnetic isolation of melanoma CTCs was reported by Freeman et al. Their study chose antigens either relatively highly expressed on melanoma cells (MCAM and MCSP) or expressed on subpopulations of melanoma cells proposed to be particularly aggressive and competent to initiate metastasis (CD271 and ACBC5). Dynabeads mediated isolation was validated with one melanoma cell line (A2058) known to express the relevant antigens, spiked into blood. Recovery efficiency was 35% with either MCAM or MCSP antibodies, with no significant increase in recovery rates by combining both antibodies. Nevertheless the authors suggest combination might still be advantageous for heterogeneous patient samples [26].

Our study revisits the same antigens, but to potentially improve on melanoma CTC isolation, tested three additional antibodies against cell surface proteins either commonly upregulated during melanoma progression (N-cadherin), or promising antigens with common membrane staining of melanoma cells (KBA.62, LHM3). To predict suitability as isolation target we analysed a cohort of eight melanoma cell lines with various genetic backgrounds and phenotypes to account for heterogeneity.

Our data confirms heterogeneous levels of MCAM expression in 6 melanoma cell lines, which is in keeping with MCAM expression in tissue of 68% of primary melanoma and 89% of melanoma lymph node metastases [27]. Very high detectable levels of MCSP were observed in 5 of 8 melanoma cell lines throughout the entire cell population, while the remaining 3 lines (501mel, MelMs and M230) lacked detectable MCSP. Targeting MCAM alone has previously shown to satisfactorily recover SkMel-28 melanoma cells (74–88% recovery) spiked into blood with the CellSearch isolation platform. In those studies the anti-MCSP antibody clone we used here for cell isolation, was not used for isolation but for identification of MCAM isolated CTCs [28, 29]. Sakaizawa et al targeted MCSP for melanoma CTC isolation with the efficiency tested on three melanoma cell lines preselected to express MCSP (888mel, 928mel or MMG1). 1–24% recovery was achieved when cells were spiked into blood of healthy donors [30]. Our data confirms that combined targeting of both MCAM and MCSP for melanoma CTC isolation using the IsoFlux CTC isolation platform improves cell isolation from cultured cells and patient blood samples. Our cultured cell isolation data also suggests that from patients with “501mel-like” CTCs, MCAM targeting would isolate CTCs. However, if in this instance relying on MCSP probing for identification, these CTCs would be missed. Importantly, in our hands the anti-MCAM antibody clone (F4-35H7) used in the CellSearch studies was similarly effective in binding melanoma cells (FACS, immunostaining and spiked cell isolation) as the more readily commercially available clone P1H12. Therefore we relied on the latter to isolate melanoma CTCs from patient blood.

Our small patient cohort includes two patients with CTCs that behaved like 501mel cells as they were not (patient 1) or relatively poorly (patient 9) isolated by MCSP but well by MCAM targeting (Table 3). More patient samples showed the opposite with higher CTC counts found with MCSP compared with MCAM based isolation. These findings support our melanoma CTC isolation strategy and combined targeting of MCAM and MCSP. This is in line with our study detecting CTCs in 87.5% of stage IV patients, while a recent study reported detection of melanoma CTCs in 42% of stage IV patients using the CellSearch strategy [31]. It should however be noted, that the combination of both antibodies not always increased CTC counts. Previously published work in ovarian cancer suggests that the presence of magnetic beads conjugated to an antibody against an antigen of minor abundance may interfere with optimum isolation of CTCs lacking the antigen [32]. Hence, use of multiple isolation antibodies for immunomagnetic cell capture, especially targeting relatively rare markers, needs careful consideration.

Regarding the other potential targets, we tested for immunomagnetic melanoma CTC isolation: N-cadherin positivity correlated with MCSP detection for all cell lines. This is not surprising since MCSP is proposed to induce epithelial to mesenchymal (EMT) like changes in melanoma cells and N-cadherin is an important EMT marker [33]. However, in all cases, a significantly lower proportion of cells (25–75%) interacted with the anti-N-cadherin antibody. Thus, although we successfully validated this antibody for isolation of EMT-CTCs from ovarian cancer patients, we disregarded it for melanoma CTC isolation in favour of the anti-MCSP antibody [32]. All tested antibodies, other than anti-MCAM and anti-MCSP, failed as relevant isolation tools for melanoma cells due to poor interaction with melanoma cells. Although some of the correlating target cell surface markers may still be expressed in some melanoma cells, since our data supports careful rationalisation of isolation antibodies, we consider targeting both MCAM and MCSP is the most viable immunomagnetic melanoma CTC isolation strategy. This is supported by other studies identifying MCAM and MCSP as dominating melanoma cell surface proteins [16].

Interestingly, disregarding marginal MCAM and LHM3 expression, both tested c-Kit mutant cell lines (MelMS and M230) were, negative for all the tested cell surface markers. This finding suggests that isolation of CTCs from c-Kit mutant melanoma patients may need further refinement. This should be kept in mind when managing patients with acral or mucosal melanomas, which have significantly higher rates of c-Kit mutation [34]. Isolation of melanoma CTCs using size-based spiral microfluidics may provide an alternative, but it is limited to cells larger than average blood cells [6].

### Detection of PD-L1 on CTCs

There is some debate regarding PD-L1 antibodies as certain antibody clones are less reliable, and others are recommended for companion diagnostics and PD-L1 detection [35]. We chose the anti-human PD-L1 rabbit monoclonal antibody (clone E1L3N), because PD-L1 positivity in tissue detected with this antibody correlates well with other anti-PD-L1 antibodies [35–37]. This antibody was also previously successfully used for PD-L1 probing in bladder cancer CTCs [38].

Although PD-L1 expression in melanomas correlates with good response to PD-1 inhibitory therapy it is not strictly required with a significant proportion of patients responding to ICI despite the lack of detectable PD-L1 in their tumor tissue [21–23]. Since PD-L1 expression in tumor tissue appears to be induced by TILs, it has been proposed that this so called adaptable PD-L1 expression in tumor cells could be the key to its value as a therapy response biomarker and adaptable PD-L1 might be missed in TIL deficient tumors [21, 35]. As predicted, our data suggest that melanoma cells in blood can show increased PD-L1 expression. Interestingly, for both BRAF mutant cell lines, recovered cell numbers did not decline but slightly increase after 24 hour storage in blood at room temperature. This might be due to effects of blood based cytokine exposure on levels of MCAM and MCSP expression, in turn improving cell isolation, combined with effective protection from anoikis by oncogenic BRAF signalling [39]. Regardless, induction of PD-L1 on melanoma cells in the blood implies that PD-L1 screening of CTCs may detect adaptable PD-L1 even when the tumor tissue exhibits no or low counts of TILs. Whether such adaptable PD-L1 on CTCs correlates with ICI response needs to be tested in suitable patient cohorts.

Unfortunately, for our proof of concept study to validate the effectiveness of our MCAM and MCSP based melanoma CTC isolation method, we had predominantly access to blood draws of patients already on ICI therapy. Nevertheless, we found overall 38.6% (5/13) of patients whose blood draws were probed for PD-L1 had some PD-L1 positive CTCs with the proportion of PD-L1 positive CTCs varying between 1.5–60%. Interestingly, although the patient cohort size is too small for any firm conclusions, there appears to be an association of PD-L1 positivity with the isolation of CTCs by targeting MCSP either alone or in combination with MCAM. This does highlight that different targeting indeed captures different populations of melanoma CTCs. If this MCSP-PD-L1 association can be confirmed in a larger patient cohort, it would be interesting to study the mechanism of presenting these two antigens together and the implication for melanoma cell recognition by immune cells.

Ideally PD-L1 assaying in CTCs should be done prior as well as during the course of treatment to evaluate their value as predictive biomarker. One would expect a higher proportion of PD-L1 positive CTCs before commencement of treatment as these cells should loose protection upon PD-1/PD-L1 inhibition and be rapidly targeted by blood based immune cells upon therapy. However, in melanoma tissue there is evidence that ISI causes increase of PD-L1 expression [40] and with CTCs being regularly replenished from tumour tissue, testing of PD-L1 on CTCs longitudinally during therapy is needed to fully understand the relationship.

Nevertheless, our PD-L1 detection on CTCs is similar to studies in other cancers. A breast cancer study using the mouse monoclonal antibody 130021 to detected PD-L1 found 69% (11/16) hormone sensitive breast cancer patients positive for CTCs (defined as ≥1 CTCs per 7.5ml blood, CellSearch) of those 72% (8/11) had PD-L1 positive CTCs with a range of 0.2–100% being PD-L1 positive [41]. With the same antibody in a non small cell lung cancer (NSCLC) study, at baseline 80% (20/25) of patients had 1–20 CTCs (Cellsearch) and of those 95% (19/20) had some CTCs with detectable PD-L1 ranging from 25–100% of the CTCs [42]. A study into PD-L1 detection on bladder cancer CTCs used the same antibody clone (E1L3N) we employed in our study. That study found CTCs in 80% of patient samples and PD-L1 positive CTCs in 35% of CTC positive patient samples. Proportion of PD-L1 positive CTCs varied between 0.9–67% [38].

Two of the mentioned studies, appraised the correlation of presence of PD-L1 positive CTC detection and ICI therapy response. In the NSCLC study by Nicolazzo et al. patient attrition meant that only 10 patients were analysed at 6 month into nivolumab treatment. There was a trend towards worse response with retention of PD-L1 positive CTCs at this time point. PD-L1 CTC detection at baseline was not response related. This was consistent with the finding that some tissue PD-L1 positive NSCLC patients were not responding to ICI [42]. Anantharaman et al’s study assessed the relationship between PD-L1 on CTCs and ICI response in only four bladder cancer patients finding progressive disease on ICI regardless of CTC PD-L1 status [38].

To our knowledge, there are no melanoma studies investigating the relationship of the presence of PD-L1 on CTCs and response to PD-1 inhibitor therapy. This is despite the widespread and expanding use of PD-1 inhibitor in melanoma therapy, currently without testing a clinically approved biomarker to predict response. More comprehensive studies are required to delineate whether PD-L1 detection on CTCs could be a suitable biomarker of ICI therapy outcomes in melanoma.

## Conclusion

We were able to develop an efficient isolation and identification protocol for melanoma CTCs which included PD-L1 detection on CTCs. Our *in vitro* data suggest that CTCs with adaptable PD-L1 may increase PD-L1 expression in the blood. Whether that makes CTCs a good biosource to screen for PD-L1 and how levels of PD-L1 on melanoma CTCs, measureable with this relatively fast and easy detection assay, correlate to patient response needs to be investigated in larger scale studies.

## Supporting information

S1 Fig Melanoma cell surface protein expression. Compiled data for all designated cell lines showing proportion of cells expressing the indicated cell surface proteins.
(TIF)

S1 Table Melanoma cell lines. List of melanoma cell lines used in this study, including genetic and phenotypic characteristics.
(DOCX)

S2 Table Candidate antibodies: Melanoma cell isolation. List of melanoma cell isolation candidate antibody details with references to melanoma association.
(DOCX)

S3 Table Immunoreactivity of melanoma cell detection antibodies. Expression of melanoma CTC identification markers in complete cohort of melanoma cell lines using non-conjugated primary antibodies.
(DOCX)
