# Supplementary figures and images for: Immunomagnetic isolation of circulating melanoma cells and detection of PD-L1 status
Source: PLoS One. 2019 Feb 8;14(2):e0211866. doi: 10.1371/journal.pone.0211866 (PMC6368301; doi:10.1371/journal.pone.0211866)

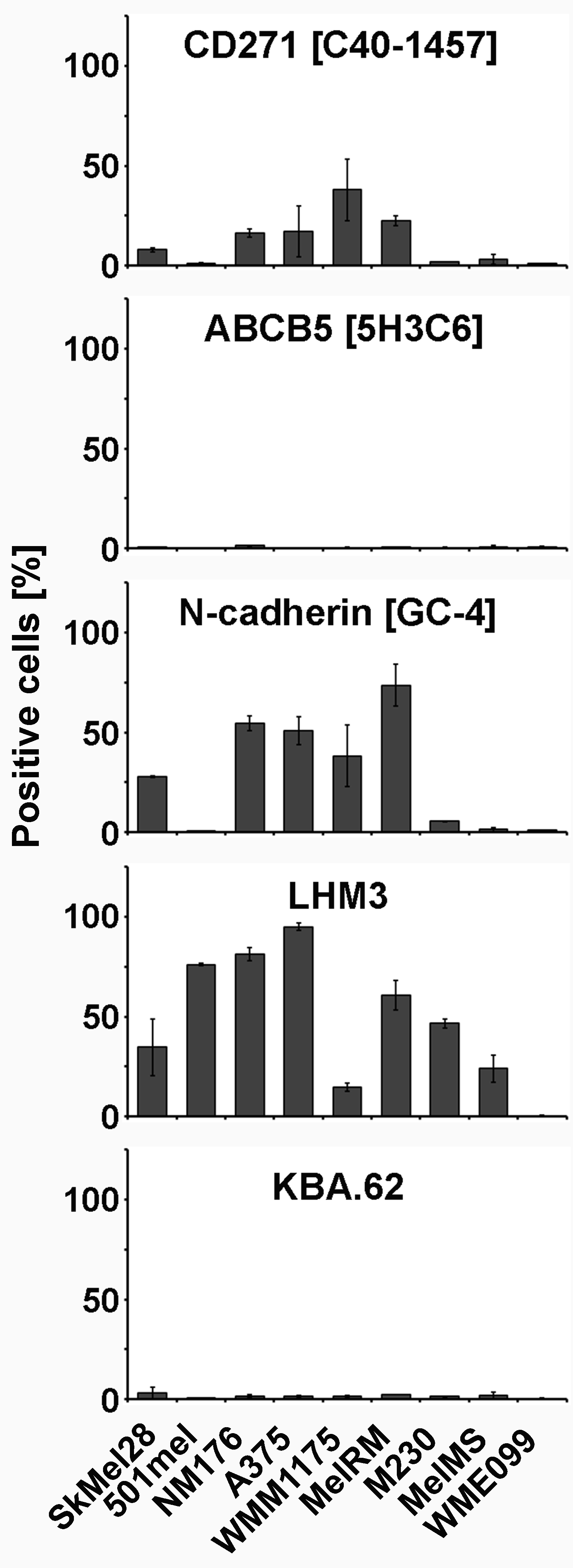

Supplement: S1 Fig — Compiled data for all designated cell lines showing proportion of cells expressing the indicated cell surface proteins. (TIF) [file pone.0211866.s001.tif]
