# Supplementary material for: Immunomagnetic isolation of circulating melanoma cells and detection of PD-L1 status
Source: PLoS One. 2019 Feb 8;14(2):e0211866. doi: 10.1371/journal.pone.0211866 (PMC6368301; doi:10.1371/journal.pone.0211866)
Supplement: S1 Table — List of melanoma cell lines used in this study, including genetic and phenotypic characteristics. (DOCX) [file pone.0211866.s002.docx]

**S1 Table** **Melanoma cell lines**

| **Cell Line** | **Mutation Signature / Characteristics** |
| --- | --- |
| **SkMel28** | BRAF^V600E^, CDK4^R24C^, EGFR^P753S^, PTEN^T167A^,PTEN^R130Q^, p53^L145R^, express wild type p16^INK4a^, epithelial expression signature [1] # |
| **501mel** | BRAF^V600E^,CTNNB1^D32H^, CTNNB1^D37F^, p16-null, [2] # |
| **NM176** | BRAF^V600E^, CDK4^R24C^, express wild type p16^INK4a^, high melanin expression [3] |
| **A375** | BRAF^V600E^, p16INK4a^E61^*, p16INK4a^E69^* # |
| **WMM1175** | NRAS^G13R^, p16-null, p53-null [4, 5] |
| **MelRM** | NRAS^Q61R^ [6] |
| **MelMS** | c-Kit^W557-K558del^, high melanin expression[7] |
| **M230** | c-Kit^L576P^ [8, 9] |

* Nonsense-substitution; # <http://cancer.sanger.ac.uk/cancergenome/projects/cosmic/>

**S1 Table References**

1. Carlino MS, Gowrishankar K, Saunders CA, Pupo GM, Snoyman S, Zhang XD, et al. Antiproliferative Effects of Continued Mitogen-Activated Protein Kinase Pathway Inhibition following Acquired Resistance to BRAF and/or MEK Inhibition in Melanoma. Molecular Cancer Therapeutics. 2013;12 1332-42. doi: 10.1158/1535-7163.MCT-13-0011. PubMed PMID: 23645591.

2. Carreira S, Goodall J, Aksan I, La Rocca SA, Galibert MD, Denat L, et al. Mitf cooperates with Rb1 and activates p21Cip1 expression to regulate cell cycle progression. Nature. 2005;433(7027):764-9. doi: 10.1038/nature03269. PubMed PMID: 15716956.

3. Rizos H, Darmanian AP, Indsto JO, Shannon JA, Kefford RF, Mann GJ. Multiple abnormalities of the p16INK4a-pRb regulatory pathway in cultured melanoma cells. Melanoma Research. 1999;9(1):10-9. PubMed PMID: 10338330.

4. Becker TM, Boyd SC, Mijatov B, Gowrishankar K, Snoyman S, Pupo GM, et al. Mutant B-RAF-Mcl-1 survival signaling depends on the STAT3 transcriptionfactor Oncogene. 2013;33(9):1158-66

5. Becker TM, Rizos H, Kefford RF, Mann GJ. Functional impairment of melanoma-associated p16(INK4a) mutants in melanoma cells despite retention of cyclin-dependent kinase 4 binding. Clinical cancer research : an official journal of the American Association for Cancer Research. 2001;7(10):3282-8. Epub 2001/10/12. PubMed PMID: 11595726.

6. Gowrishankar K, Gunatilake D, Gallagher SJ, Tiffen J, Rizos H, Hersey P. Inducible but not constitutive expression of PD-L1 in human melanoma cells is dependent on activation of NF-kappaB. PloS one. 2015;10(4):e0123410. doi: 10.1371/journal.pone.0123410. PubMed PMID: 25844720; PubMed Central PMCID: PMCPMC4386825.

7. Todd JR, Scurr LL, Becker TM, Kefford RF, Rizos H. The MAPK pathway functions as a redundant survival signal that reinforces the PI3K cascade in c-Kit mutant melanoma. Oncogene. 2014;33(2):236-45. Epub 2012/12/19. doi: 10.1038/onc.2012.562. PubMed PMID: 23246970.

8. Todd JR, Becker TM, Kefford RF, Rizos H. Secondary c-Kit mutations confer acquired resistance to RTK inhibitors in c-Kit mutant melanoma cells. Pigment Cell & Melanoma Research. 2013;26:518-26. doi: 10.1111/pcmr.12107. PubMed PMID: 23582185.

9. von Euw E, Atefi M, Attar N, Chu C, Zachariah S, Burgess BL, et al. Antitumor effects of the investigational selective MEK inhibitor TAK733 against cutaneous and uveal melanoma cell lines. Molecular Cancer. 2012;11:22. doi: 10.1186/1476-4598-11-22. PubMed PMID: 22515704; PubMed Central PMCID: PMC3444881.
