# Supplementary material for: Immunomagnetic isolation of circulating melanoma cells and detection of PD-L1 status
Source: PLoS One. 2019 Feb 8;14(2):e0211866. doi: 10.1371/journal.pone.0211866 (PMC6368301; doi:10.1371/journal.pone.0211866)
Supplement: S2 Table — List of melanoma cell isolation candidate antibody details with references to melanoma association. (DOCX) [file pone.0211866.s003.docx]

**S2 Table** **Candidate antibodies: melanoma cell isolation**

| **Antigen [other names]** | **Clone, distributer** | **Concentration** | **Comments** |
| --- | --- | --- | --- |
| **MCAM** (melanoma cell adhesion molecule), [MelCAM, MUC18, ***CD146***/ S-Endo, A32] | P1H12, Millipore | IF/FACS: 1:250  IM: 1µg | Marker of melanoma progression [1]; used for melanoma CTC isolation with Dynabeads [2] |
|  | F4-35H7, Biocytex | IF/FACS: 1:20  IM: 1:4^§^ | Used for melanoma CTC isolation with CellSearch instrument [3-5] |
| **MCSP** (melanoma chondroitin sulfate proteoglycan)**,** [high molecular weight-melanoma associated antigen (HMW-MAA), neuron-glial antigen 2 (NG2)] | 9.2.27^#^, Millipore | IF/FACS: 1:500  IM: 0.5µg | MSCP reviewed by [6]; used for melanoma CTC isolation with Dynabeads [2] |
| **CD271** [nerve growth factor receptor p75NGFR, p75NTR, LNGFR] | C40-1457, BD Biosciences | IF/FACS: 1:500  IM: 0.5µg | Proposed as marker for aggressive, metastatic melanoma cells within heterogeneous populations [7]; used for melanoma cell isolation with Dynabeads [2] |
| **ABCB5**  (ATP-binding cassette protein) | 5H3C6, Abcam | IF/FACS: 1:100  IM: N/A | Proposed as marker for aggressive, metastatic melanoma cells within heterogeneous populations [8]; *different rabbit anti-ABCB5* used for melanoma CTC isolation with Dynabeads [2] |
| **N-cadherin** [***CD325***] | GC-4, Abcam | IF/FACS: 1:20  IM: 1.25µg | Increases with melanoma progression [9]; used for ovarian cancer CTC isolation [10] |
| **LHM3** | LHM3, Santa Cruz | IF/FACS: 1:40  IM: N/A | Anti-melanoma antibody raised against A375 extract; candidate for imaging of disseminated melanoma cells [11] |
| **KBA.62** “” | KBA.62, Abcam, | IF/FACS: 1:40  IM: N/A | Anti-melanoma antibody raised against KAL extract; melanoma specific membrane antigen [12] |

#: mouse IgG2a, all other antibodies are mouse IgG1; IF/FACS: antibody dilution used for immunofluorescence and FACS analysis; IM: antibody amount used to conjugate 50µl of magnetic beads; ^§^ no concentration provided; N/A not applicable

**S2 Table References**

1. Pearl RA, Pacifico MD, Richman PI, Wilson GD, Grover R. Stratification of patients by melanoma cell adhesion molecule (MCAM) expression on the basis of risk: implications for sentinel lymph node biopsy. Journal of Plastic, Reconstructive & Aesthetic Surgery. 2008;61(3):265-71. doi: 10.1016/j.bjps.2007.04.010. PubMed PMID: 17569608.

2. Freeman JB, Gray ES, Millward M, Pearce R, Ziman M. Evaluation of a multi-marker immunomagnetic enrichment assay for the quantification of circulating melanoma cells. Journal of Translational Medicine. 2012;10:192. doi: 10.1186/1479-5876-10-192. PubMed PMID: 22978632; PubMed Central PMCID: PMC3480925.

3. Rao C, Bui T, Connelly M, Doyle G, Karydis I, Middleton MR, et al. Circulating melanoma cells and survival in metastatic melanoma. International Journal of Oncology. 2011;38(3):755-60. doi: 10.3892/ijo.2011.896. PubMed PMID: 21206975.

4. Khoja L, Lorigan P, Zhou C, Lancashire M, Booth J, Cummings J, et al. Biomarker Utility of Circulating Tumor Cells in Metastatic Cutaneous Melanoma. The Journal of Investigative Dermatology. 2012. doi: 10.1038/jid.2012.468. PubMed PMID: 23223143.

5. Hall CS, Ross M, Bowman Bauldry JB, Upshaw J, Karhade MG, Royal R, et al. Circulating Tumor Cells in Stage IV Melanoma Patients. J Am Coll Surg. 2018;227(1):116-24. doi: 10.1016/j.jamcollsurg.2018.04.026. PubMed PMID: 29746918.

6. Campoli MR, Chang CC, Kageshita T, Wang X, McCarthy JB, Ferrone S. Human high molecular weight-melanoma-associated antigen (HMW-MAA): a melanoma cell surface chondroitin sulfate proteoglycan (MSCP) with biological and clinical significance. Crit Rev Immunol. 2004;24(4):267-96. PubMed PMID: 15588226.

7. Boiko AD, Razorenova OV, van de Rijn M, Swetter SM, Johnson DL, Ly DP, et al. Human melanoma-initiating cells express neural crest nerve growth factor receptor CD271. Nature. 2010;466(7302):133-7. doi: 10.1038/nature09161. PubMed PMID: 20596026; PubMed Central PMCID: PMC2898751.

8. Schatton T, Murphy GF, Frank NY, Yamaura K, Waaga-Gasser AM, Gasser M, et al. Identification of cells initiating human melanomas. Nature. 2008;451(7176):345-9. doi: 10.1038/nature06489. PubMed PMID: 18202660; PubMed Central PMCID: PMC3660705.

9. Watson-Hurst K, Becker D. The role of N-cadherin, MCAM and beta3 integrin in melanoma progression, proliferation, migration and invasion. Cancer Biology & Therapy. 2006;5(10):1375-82. PubMed PMID: 16969099.

10. Po JW, Roohullah A, Lynch D, DeFazio A, Harrison M, Harnett PR, et al. Improved ovarian cancer EMT-CTC isolation by immunomagnetic targeting of epithelial EpCAM and mesenchymal N-cadherin. Journal of Circulating Biomarkers. 2018;7:184945441878261. doi: 10.1177/1849454418782617.

11. Kupsch JM, Tidman N, Bishop JA, McKay I, Leigh I, Crowe JS. Generation and selection of monoclonal antibodies, single-chain Fv and antibody fusion phage specific for human melanoma-associated antigens. Melanoma Research. 1995;5(6):403-11. PubMed PMID: 8589614.

12. Cohen-Knafo E, al Saati T, Aziza J, Ralfkiaer E, Selves J, Gorguet B, et al. Production and characterisation of an antimelanoma monoclonal antibody KBA.62 using a new melanoma cell line reactive on paraffin wax embedded sections. Journal of Clinical Pathology. 1995;48(9):826-31. PubMed PMID: 7490315; PubMed Central PMCID: PMC502870.
