# Supplementary material for: Immunomagnetic isolation of circulating melanoma cells and detection of PD-L1 status
Source: PLoS One. 2019 Feb 8;14(2):e0211866. doi: 10.1371/journal.pone.0211866 (PMC6368301; doi:10.1371/journal.pone.0211866)
Supplement: S3 Table — Expression of melanoma CTC identification markers in complete cohort of melanoma cell lines using non-conjugated primary antibodies. (DOCX) [file pone.0211866.s004.docx]

**S3 Table** **Immunoreactivity of melanoma cell detection antibodies**

| **Cell line** | **MelanA** | **S100-b** | **Gp100** | **All combined** |
| --- | --- | --- | --- | --- |
| **SkMel28** | ++ | +++ | +++ | +++ |
| **501mel** | +++ | +/- | ++ | +++ |
| **NM176** | +++ | ++(-) | ++ | +++ |
| **A375** | - | +- | ++ | ++ |
| **WMM1175** | - | +- | ++ | ++ |
| **MelRM** | +/- | ++ | +/- | ++ |
| **MelMS** | +++ | ++ | +++ | +++ |
| **M230** | +++ | ++ | +++ | +++ |

+++ very strong in all cells, ++ strong in >85% of cells, + clearly detectable in most cells,+/ - clearly detectable in >40% of cells, - negative
